# Association Between Plasma Granzyme B Levels, Organ Failure, and 28-Day Mortality Prediction in Patients with Sepsis

**DOI:** 10.3390/jcm14051461

**Published:** 2025-02-21

**Authors:** Min Seo Ki, Ju Hye Shin, Min Dong Sung, Shihwan Chang, Ah Young Leem, Su Hwan Lee, Moo Suk Park, Young Sam Kim, Kyung Soo Chung

**Affiliations:** 1 Division of Pulmonary and Critical Care Medicine, Department of Internal Medicine, Severance Hospital, Yonsei University College of Medicine, Seoul 03722, Republic of Korea; mski9@nhimc.or.kr (M.S.K.);; 2 Division of Pulmonology, Department of Internal Medicine, National Health Insurance Service Ilsan Hospital, Goyang 10444, Republic of Korea

**Keywords:** sepsis, prognosis, granzyme B, mortality prediction, lactate, SOFA

## Abstract

**Background/Objectives**: Sepsis is basically an inflammatory disease that involves the host’s immune response. Granzyme B, a cytotoxic protease, has garnered attention for its involvement in modulating immune responses. This study aimed to elucidate the clinical implications of granzyme B in critically ill patients with sepsis, focusing on plasma granzyme B levels as a potential prognostic marker. **Methods**: We conducted a retrospective analysis of sequentially collected blood samples from 57 sepsis patients admitted to the medical intensive care unit at Severance Hospital, a tertiary hospital in Seoul, South Korea. Clinical and laboratory data were comparatively analyzed between 28-day survivors and nonsurvivors. **Results**: The number of patients in the survivor and nonsurvivor groups was 32 (56.1%) and 25 (43.9%), respectively. Compared to survivors, nonsurvivors had higher APACHE II (23.5 vs. 34, *p* = 0.007) and SOFA (10 vs. 15, *p* = 0.001) scores, as well as increased levels of serum lactate (1.8 vs. 9.2 mmol/L, *p* < 0.001) and plasma granzyme B (28.2 vs. 71 pg/mL, *p* < 0.001). Granzyme B exhibited a robust area under the receiving operating characteristic (AUROC) for predicting 28-day mortality (AUROC = 0.794), comparable to lactate (0.804), SOFA (0.764), and APACHE II (0.709). The combined index of lactate and granzyme B demonstrated the highest AUROC (0.838) among all investigated predictors. Significant positive correlations were observed between log granzyme B and various inflammatory cytokines, including log IFN-γ (r = 0.780), IL-4 (r = 0.540), IL-10 (r = 0.534), and IL-6 (r = 0.520). **Conclusions**: Plasma granzyme B demonstrated fair short-term mortality prediction among patients admitted to the ICU, suggesting its potential utility for risk stratification and managing patients with sepsis.

## 1. Introduction

Sepsis remains a formidable challenge in critical care medicine, with affected patients exhibiting high morbidity and mortality rates, necessitating the identification of reliable prognostic markers to guide clinical management and optimize patient care [1,2]. In recent years, emerging evidence has implicated immune dysregulation in the pathophysiology of sepsis, shedding light on the potential role of various immune molecules as prognostic markers [3,4].

Granzyme B, a serine protease primarily associated with cytotoxic T lymphocytes and natural killer cells [5,6], has garnered attention for its involvement in modulating immune responses. While studies have explored its role in various inflammatory conditions [7,8], its significance in the context of sepsis, particularly in critically ill patients admitted to the intensive care unit (ICU), remains insufficiently elucidated [9]. Few studies have evaluated the clinical utility of granzyme B compared to existing biomarkers, its correlation with inflammatory mediators and organ failure indicators, or its trends following treatment using clinical data.

This study evaluated the significance of granzyme B as a short-term prognostic predictor in medical ICU patients with confirmed sepsis, comparing it with established biomarkers. Additionally, we analyzed its trends during the first week of ICU admission and investigated its correlation with pro-inflammatory cytokines, lactate, and SOFA score to further elucidate its role in sepsis.

## 2. Materials and Methods

### 2.1. Study Design and Patient Population

This study involved retrospective analysis of the prospectively collected blood samples from patients admitted to the medical intensive care unit (MICU) at Severance Hospital, a 2000-bed (30-bed medical ICU) university tertiary referral hospital in Seoul, South Korea. The study protocol was approved by the Institutional Review Board (IRB) of Severance Hospital (IRB number 4-2019-1230). Written informed consent was obtained from the patients or their families. All study procedures were performed in accordance with the relevant guidelines and regulations. In addition, this study was performed in compliance with the principles set forth in the Declaration of Helsinki.

All patients aged ≥ 18 years with suspected sepsis admitted to the MICU were eligible to be included in the MICU sepsis cohort. Among 119 consecutive patients in the MICU sepsis cohort, patients diagnosed with sepsis according to the Sepsis-3 guideline were included in this study. After excluding patients for whom 28-day mortality data were missing, the study ultimately included 57 patients with confirmed sepsis. These patients were categorized into survivors and nonsurvivors based on their outcomes after 28 days of ICU admission.

### 2.2. Definitions

Sepsis was defined as a condition with life-threatening organ dysfunction owing to a dysregulated host response to infection. The sequential organ failure assessment (SOFA) score is used to assess organ dysfunction [10], and an increase of ≥2 points in the SOFA score in patients with suspected infection is indicative of sepsis.

### 2.3. Data Collection

The following clinical data were collected: age, sex, Charlson comorbidity index (CCI) score, infection focus, presence of bacteremia, and bacteremic pathogen. Vital signs, laboratory tests, length of stay, and 28-day survival data were electronically extracted from the hospital database. The acute physiology and chronic health evaluation II (APACHE II) and SOFA scores were determined using data obtained within 24 h of ICU admission. Biomarker levels, including procalcitonin, C-reactive protein, and lactate, along with white blood cell counts, were obtained from routine laboratory tests conducted upon ICU admission. The use of vasopressors, mechanical ventilation, continuous renal replacement therapy (CRRT), and extracorporeal membrane oxygenation (ECMO) was determined based on their application within the first week after ICU admission. Data on microbiological etiology, including bacteremia and bacteremic pathogens, were collected from records within two days before and after ICU admission.

### 2.4. Blood Sample Collection and Measurement of Plasma Granzyme B

Venous whole blood was collected on the day of admission to the ICU (time point 0) and subsequently on day 1 (time point 1), day 3 (time point 3), and day 7 (time point 7) for as long as the patients remained alive and stayed in the ICU. The distribution of the collected samples varied among the patients as follows: 37 patients had samples collected at all four time points (time points 0, 1, 3, and 7); 8 patients had samples collected at time points 0, 1, and 3; and 12 patients had samples collected at time points 0 and 1. Venous blood samples were collected through central lines into tubes containing ethylenediaminetetraacetic acid. Plasma was prepared by centrifugation at 800× *g* for 15 min at 4 °C. Supernatants from centrifuged blood were immediately aliquoted and stored at –80 °C until analysis. Plasma levels of granzyme B and other cytokines [tumor necrosis factor (TNF)-α, interleukin (IL)-6, IL-10, IL-4, IL-2, IL-1β/IL-1F2, IL-18/IL-1F4, chemokine ligand 2/monocyte chemoattractant protein 1 (CCL2/MCP-1), interferon-gamma (IFN-γ)] were analyzed using the Human Magnetic Luminex^®^ Screening Assay kit (R&D Systems, Inc., Minneapolis, MN, USA). All samples and standards were assayed in duplicate using the Luminex 200TM System (Merck Millipore, Darmstadt, Germany).

### 2.5. Statistical Analysis

Clinical parameters were analyzed using the Mann–Whitney U test for continuous variables and the Chi-square test or Fisher’s exact test for categorical variables. Continuous variables are expressed as the median with interquartile range, and categorical variables are expressed as the number with percentage. 

The predictive performance for 28-day mortality was assessed using the area under the receiver operating characteristic curve (AUROC), with comparisons between AUROCs conducted using the DeLong test. Patients were categorized into high- and low-granzyme B groups based on the optimal cutoff value determined by Youden’s index. Kaplan–Meier survival curves were generated for both groups, with survival estimates calculated using the Kaplan–Meier method and compared using the log-rank test. Univariable and multivariable Cox proportional hazards regression analyses were performed to identify independent predictors of 28-day mortality. Pearson correlation coefficients (r) were calculated to investigate the relationship between plasma granzyme B and various cytokines and SOFA scores. *p*-values less than 0.05 were considered statistically significant. The statistical analyses were performed using R Statistical Software, version 3.4.1 (The R Foundation for Statistical Computing, Vienna, Austria).

## 3. Results

**Figure 1:**
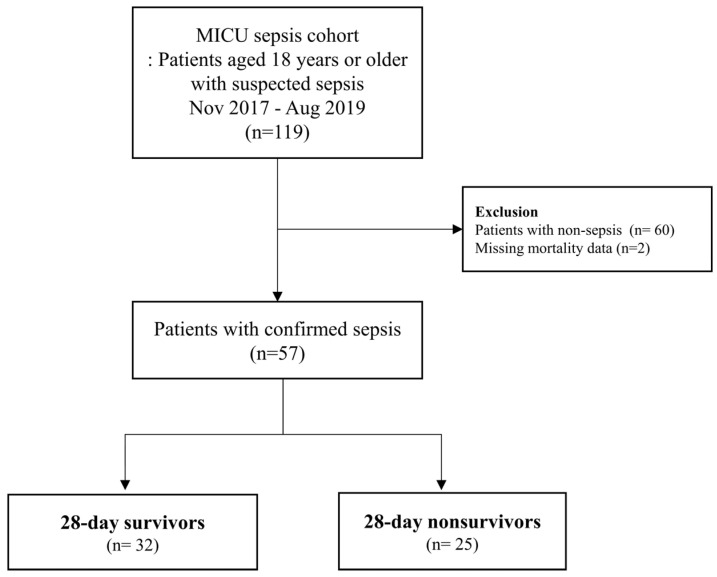
Flowchart of the study patient selection process.

The patients were classified as 28-day survivors and 28-day nonsurvivors according to mortality on the 28th day of ICU admission. The number of patients in the survivor and nonsurvivor groups was 32 (56.1%) and 25 (43.9%), respectively (Figure 1).

Table 1 presents the clinical characteristics distinguishing 28-day survivors and 28-day nonsurvivors. The 28-day nonsurvivors exhibited higher CCI scores (4 vs. 6; *p* = 0.004) and a greater incidence of concurrent malignant diseases than 28-day survivors (18.8% vs. 48%; *p* = 0.038). In both groups, the primary origin of infection was found in the pulmonary region, and no significant statistical differences were observed in the incidence of bacteremia (*p* = 0.573) and shock (*p* = 1.000). Compared to 28-day survivors, 28-day nonsurvivors had higher APACHE II (23.5 vs. 34, *p* = 0.007) and SOFA (10 vs. 15, *p* = 0.001) scores, as well as increased levels of serum lactate (1.8 vs. 9.2 mmol/L, *p* < 0.001) and plasma granzyme B (28.2 vs. 71 pg/mL, *p* < 0.001). No significant differences in white blood cell count (*p* = 0.539), C-reactive protein (*p* = 0.053), procalcitonin (*p* = 0.333), and IL-6 (*p* = 0.186) levels were observed between the two groups. Compared to 28-day survivors, 28-day nonsurvivors required a greater number of vasopressors (*p* = 0.016) and had significantly higher peak norepinephrine infusion rates on the day of ICU admission (*p* = 0.001). Additionally, the incidence of acute kidney injury (*p* = 0.037) and the rate of CRRT implementation (*p* = 0.001) were higher in 28-day nonsurvivors.

**Figure 2:**
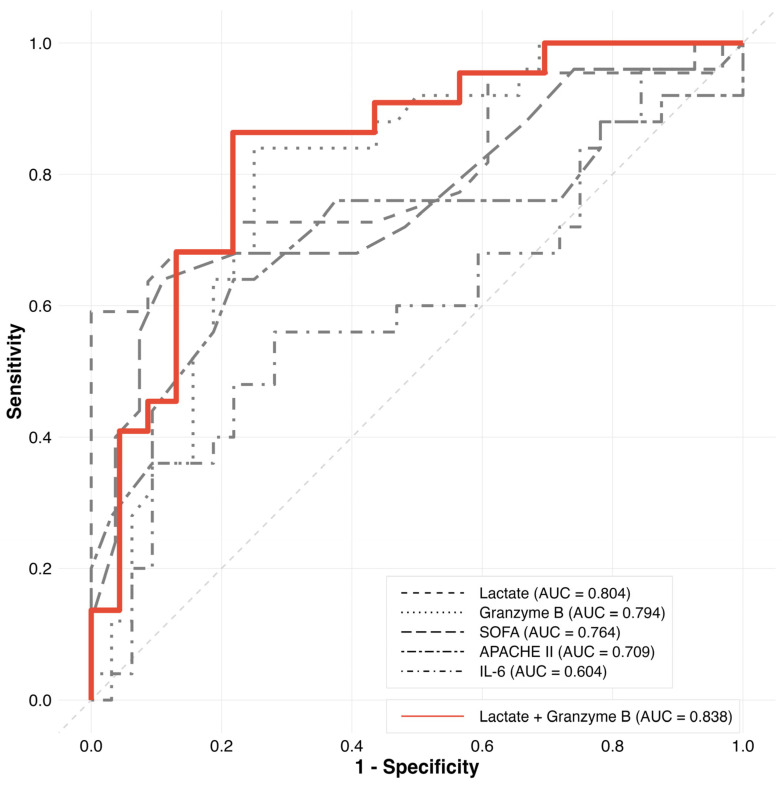
Area under the receiver operating characteristics curve for 28-day mortality of the study participants.

The SOFA and APACHE II scores, as well as lactate and IL-6 levels, were identified as prognostic factors in patients with sepsis [10,11,12,13]. The ability of these factors, including plasma granzyme B, to predict 28-day mortality in patients with sepsis was statistically significant, except for IL-6. Granzyme B demonstrated a robust AUROC of 0.794, comparable to lactate’s AUROC of 0.804. Compared to granzyme B, SOFA and APACHE II scores exhibited smaller AUROC values of 0.764 and 0.709, respectively. However, these differences in AUROCs between granzyme B levels, SOFA, and APACHE II scores were not statistically significant. The combined index of lactate and granzyme B demonstrated the highest AUROC among all investigated predictors (Figure 2 and Table 2).

**Figure 3:**
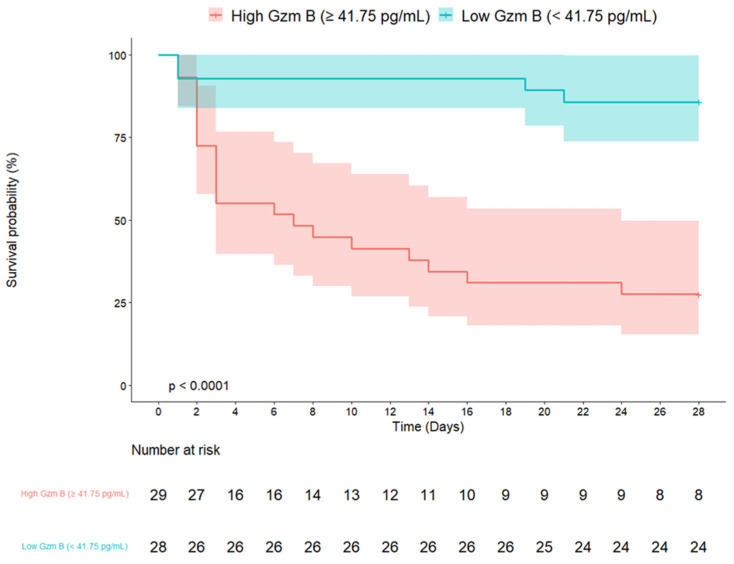
Kaplan–Meier curves for 28-day survival rate in patients with sepsis: High-Gzm B group vs. Low-Gzm B group.

The survival curves for the two groups, stratified by the granzyme B cutoff value (41.75 pg/mL) based on its predictive ability for 28-day mortality, are inherently expected to show a significant difference. As shown in Figure 3, this separation between the groups was confirmed (log-rank test, *p* < 0.001). In a multivariable Cox proportional hazards regression analysis for 28-day mortality, granzyme B levels ≥ 41.75 pg/mL were independently associated with increased mortality after adjusting for age, CCI, and SOFA (hazard ratio = 4.47; 95% confidence interval [CI] = 1.29–15.44; *p* = 0.018) (Table 3).

### 3.1. Serial Trend of Plasma Granzyme B and SOFA Score

**Figure 4:**
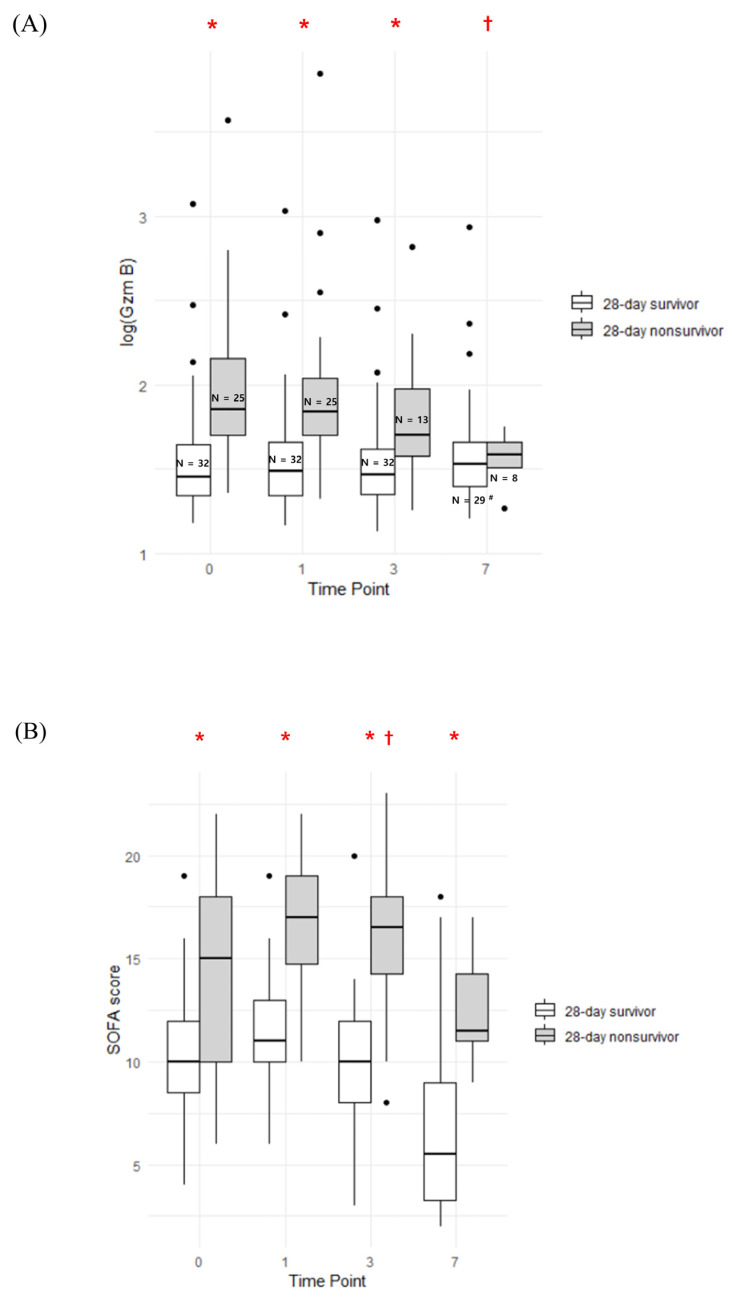
Serial trend of plasma granzyme B (**A**) and SOFA score (**B**). (**A**) * *p* < 0.05 for the difference in log_10_(Granzyme B) in 28-day survivors vs. 28-day nonsurvivors at a specified time point. † *p* < 0.05, as analyzed by generalized estimating equations, for the change in log_10_(Granzyme B) from time point 0 to a specified time point in 28-day survivors vs. 28-day nonsurvivors. ^#^ There were 3 cases of missing blood samples. (**B**) * *p* < 0.05 for the difference in SOFA score in 28-day survivors vs. 28-day nonsurvivors at a specified time point. † *p* < 0.05, as analyzed by generalized estimating equations, for the change in SOFA score from time point 0 to a specified time point in 28-day survivors vs. 28-day nonsurvivors.

From time point 0 to 3, higher granzyme B levels were observed in nonsurvivors than in survivors. Granzyme B levels in nonsurvivors, which were generally elevated, tended to decrease during ICU stay, but survivors maintained a level similar to the baseline level. In their SOFA score trends, nonsurvivors showed a remarkably elevated SOFA score compared to survivors at all time points. By time point 3, there was a tendency for the SOFA score to increase in nonsurvivors compared to time point 0, whereas this trend remained relatively stable in survivors (Figure 4).

### 3.2. Correlation Analysis Between Plasma Granzyme B and Various Cytokines

**Figure 5:**
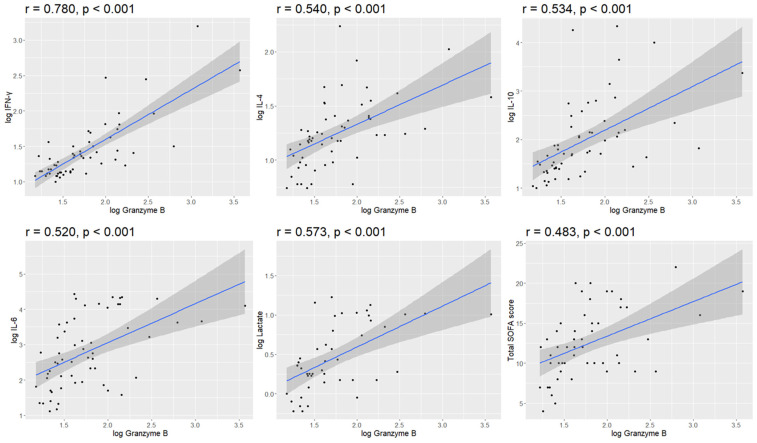
Correlation analysis between plasma granzyme B and inflammatory cytokines, lactate, and SOFA score. Scatter plots illustrate the Pearson correlations between log-transformed granzyme B and various inflammatory cytokines (IFN-γ, IL-4, IL-10, and IL-6), as well as lactate and the SOFA score. Each dot represents an individual data point. The solid regression line depicts the fitted linear relationship between the variables, while the shaded area represents the 95% confidence interval. The Pearson correlation coefficient (r) and its corresponding *p*-value are displayed in each plot.

Significant positive correlations were observed between log granzyme B and various inflammatory cytokines, including IFN-γ (r = 0.780), IL-4 (r = 0.540), IL-10 (r = 0.534), and IL-6 (r = 0.520). Additionally, log granzyme B exhibited fair correlations with lactate (r = 0.573) and the SOFA score (r = 0.483) (Figure 5). However, no significant correlations were observed between log granzyme B and C-reactive protein or procalcitonin.

## 4. Discussion

The present study revealed that plasma granzyme B levels were elevated in sepsis patients with poor 28-day survival and independently associated with short-term mortality, regardless of age, CCI, and SOFA score. The predictive performance of plasma granzyme B for 28-day mortality was comparable to that of lactate and the SOFA score, with the combined marker of granzyme B and lactate demonstrating superior predictive accuracy. Plasma granzyme B showed correlations with various inflammatory cytokines, lactate, and SOFA score as well. In 28-day nonsurvivors, a trend of decreasing plasma granzyme B levels during treatment was observed, distinct from survivors, highlighting the greater clinical significance of baseline granzyme B levels in sepsis prognosis compared to short-term changes following treatment.

Sepsis is marked by a dysregulated host response to infection, with the specific mechanisms of cell injury and organ dysfunction remaining incompletely understood [14]. It is an inflammatory disease driven by the innate immune system [15].

Granzymes, primarily located in cytotoxic lymphocytes, play a dual role in inducing cell death and mediating infection and inflammation. While their involvement in eliminating infected or abnormal cells is well-documented, they also contribute to inflammation, cytokine release, and various sepsis-related alterations [16,17,18].

In sepsis, various immune cell types, such as NK cells, T cells, mast cells, and platelets, release granzymes upon activation during infection. Studies including patients with sepsis revealed elevated plasma levels of granzymes A and B, coupled with their increased intracellular expression in cytotoxic T lymphocytes [16,19,20].

The mechanism through which granzyme B is associated with sepsis is as follows: Granzyme B influences cytokine activation by cleaving IL-1α precursors, enhancing its pro-inflammatory effects [16,21]. Additionally, granzyme B interacts with coagulation-related proteins, such as von Willebrand factor (VWF) or fibrinogen, potentially contributing to microthrombus formation and inflammatory mediator release [22]. During sepsis, platelets, a crucial player in sepsis pathophysiology, can express granzyme B, which may contribute to endothelial dysfunction and immune dysregulation, further exacerbating disease severity [23,24]. Moreover, granzyme-mediated cell death mechanisms, including apoptosis and pyroptosis, are linked to organ dysfunction in sepsis. Upon entering target cells, granzyme B activates caspase-3 and other apoptotic pathways, contributing to tissue damage and multi-organ failure [6]. Lastly, granzyme B degrades endothelial tight junction proteins, increasing capillary permeability, tissue edema, and hypotension, further contributing to organ failure [25]. These findings suggest that granzyme functions as a key regulator in the cytokine network, playing a role in the complex cytokine storm observed in sepsis.

Despite granzyme B’s potential significance, research on the prognostic value of granzymes in patients with sepsis is limited. Recently, an association has been reported between high serum granzyme B concentration and high mortality rate in patients with sepsis after controlling for diabetes mellitus, sepsis-related organ failure assessment, lactic acid level, and age [9]. Our investigation demonstrated that blood granzyme B concentrations exhibit fair predictive capabilities for 28-day mortality, consistent with the prior finding [9]. The higher optimal cutoff value for granzyme B in our study is attributed to the higher severity of sepsis in the study patients (SOFA score of nonsurvivors; 12 in [9] vs. 15 in this study). This study revealed that granzyme B level exhibited a predictive ability for 28-day mortality comparable to that of other well-established prognostic factors in patients with sepsis, such as lactate and SOFA score [10,26].

Granzymes B has previously demonstrated a correlation with various inflammatory cytokines, including IFN-γ, IL-4, and IL-10, in mouse and human sample studies [27,28,29,30], consistent with the findings of this study. The observed association between granzyme B and inflammatory cytokines supports the notion that granzyme B plays a role in inflammatory responses. This finding underscores the broader function of granzyme B beyond cytotoxicity, as it is secreted by different immune cells and contributes to cytokine regulation in inflammatory reactions. Understanding the role of granzyme B in inflammatory responses in patients with sepsis has significant implications for its potential as a prognostic marker and novel therapeutic approach.

While baseline cytokine levels can offer insights into the prognosis of patients with sepsis, changes in markers during treatment may provide more meaningful predictions for patient outcomes [31]. We investigated the clinical relevance of the temporal trend of granzyme B, serving as a functional marker of cytotoxic T cells and an inflammatory marker. Notably, a substantial decrease in granzyme B levels on the seventh day after ICU admission was observed among 28-day nonsurvivors. In contrast, survivors did not demonstrate significant alterations in granzyme B levels, likely attributable to baseline-level differences. This suggested that baseline granzyme B holds greater clinical significance in sepsis prognosis than short-term changes following treatment.

The trend of decline in inflammatory biomarkers in sepsis may indicate either effective treatment or immune exhaustion. Sepsis-associated immune suppression is characterized by elevated anti-inflammatory cytokines, depletion and apoptosis of immune cells, and T cell exhaustion [32,33,34]. Granzyme B can contribute significantly by inducing apoptosis and pyroptosis, depleting immune cells, and promoting the upregulation of inhibitory receptors, impairing their function and cytokine production. This may exacerbate immune dysregulation and enhance overall immune suppression in sepsis. Future research is needed to explore granzyme B’s role in immune exhaustion during sepsis and investigate strategies to inhibit or modulate granzyme B to restore immune function and improve outcomes.

Granzyme B has potential as a biomarker for immune dysregulation in sepsis, reflecting both inflammation and immune cell apoptosis. Combined with established biomarkers like lactate and IL-6, a multi-biomarker approach could enhance sepsis management by distinguishing between active inflammation and immune exhaustion. This could guide decisions on escalating or de-escalating immunosuppressive therapies [35].

This study has several limitations. First, the generalizability of the findings may be limited, as it was a single-center study with a small cohort and no validation cohort. Future research regarding a validation study using an independent cohort or a larger sample size would enhance the robustness of these findings. Moreover, the severity of sepsis in patients admitted to the ICU was presumably higher in this study than in previous studies. Future studies incorporating a larger and more diverse group of patients with varying severity degrees of sepsis are warranted to better understand the role of granzyme B as a prognostic marker. Second, this study examined blood samples only from the MICU sepsis cohort, and patients with sepsis treated in the emergency department or general ward were excluded from the analysis. Additionally, healthy controls were not included to compare their plasma granzyme B levels with those of patients with sepsis. Finally, a temporal gap existed between the collection of blood samples from the cohort and analysis, potentially leading to protein degradation. Nevertheless, the blood samples were promptly aliquoted and stored in a refrigerator at −80 °C to mitigate this issue. Additionally, the samples were thawed only once for the analysis.

In conclusion, plasma granzyme B demonstrated fair short-term mortality prediction among patients with sepsis in the ICU, suggesting its potential utility for risk stratification and managing patients with sepsis.

## Figures and Tables

**Table 1:** Clinical characteristics of the study patients: Comparisons of 28-day survivors and 28-day nonsurvivors.

	28-Day Survivors (N = 32)	28-Day Nonsurvivors (N = 25)	*p*-Value
Age	69.0 [63.0; 79.5]	69.0 [65.0; 81.0]	0.693
Sex, male, n (%)	20 (62.5%)	16 (64.0%)	1.000
Charlson comorbidity index	4.0 [2.0; 5.5]	6.0 [4.0; 7.0]	0.004
Immunocompromised or autoimmune disease, n (%)			
Malignancy	6 (18.8%)	12 (48.0%)	0.038
Solid organ transplantation	5 (15.6%)	2 (8.0%)	0.643
Autoimmune disease	1 (3.2%)	0 (0.0%)	1.000
Infection focus, n (%)			0.283
-Pulmonary	13 (50.0%)	14 (63.6%)	
-Urogenital	7 (26.9%)	1 (4.5%)	
-GI tract	2 (7.7%)	2 (9.1%)	
-Skin, Soft tissue, and Bone	0 (0.0%)	2 (9.1%)	
-Cardiac	1 (3.8%)	1 (4.5%)	
-Brain/CSF	0 (0.0%)	1 (4.5%)	
-Miscellaneous	1 (3.8%)	0 (0.0%)	
-Multiple	2 (7.7%)	1 (4.5%)	
Bacteremia, n (%)	12 (38.7%)	12 (50.0%)	0.573
Bacteremia pathogen, n (%)			0.587
-Gram-negative	8 (66.7%)	7 (58.3%)	
-Gram-positive	4 (33.3%)	4 (33.3%)	
-Fungal	0 (0.0%)	1 (8.3%)	
Shock, n (%)	28 (87.5%)	22 (88.0%)	1.000
APACHE II score	23.5 [19.0; 30.5]	34.0 [27.0; 39.0]	0.007
Total SOFA score	10.0 [8.5; 12.0]	15.0 [10.0; 18.0]	0.001
Use of vasopressor	31 (96.9%)	25 (100.0%)	1.000
Number of different vasopressors			0.016
0	1 (3.1%)	0 (0.0%)	
1	21 (65.6%)	6 (24.0%)	
2	9 (28.1%)	17 (68.0%)	
3	1 (3.1%)	1 (4.0%)	
4	0 (0.0%)	1 (4.0%)	
Peak norepinephrine infusion rate (mcg/kg/min) *	0.3 ± 0.3	0.8 ± 0.6	0.001
Acute kidney injury	25 (78.1%)	25 (100.0%)	0.037
CRRT	13 (40.6%)	22 (88.0%)	0.001
Mechanical ventilation	20 (62.5%)	22 (88.0%)	0.062
ECMO	3 (9.4%)	0 (0.0%)	0.329
Laboratory tests			
White blood cell count (10^3^/uL)	13.3 [11.3; 17.8]	11.0 [7.1; 23.9]	0.539
Procalcitonin (ng/mL)	5.5 [0.7; 19.4]	11.5 [2.0; 19.7]	0.333
C-reactive protein (mg/L)	189.4 [99.2; 311.1]	140.2 [49.2; 201.5]	0.053
Lactate, venous (mmol/L)	1.8 [0.9; 2.4]	9.2 [1.8; 10.6]	<0.001
Granzyme B (pg/mL)	28.2 [21.8; 46.8]	71.0 [50.5; 143.5]	<0.001
Interleukin-6 (pg/mL)	353.1 [89.6; 1605.0]	1282.2 [88.5; 12,950.0]	0.186

Data are shown as median with interquartile range or n (%). CSF, cerebrospinal fluid; GI, gastrointestinal tract; APACHE II, acute physiology and chronic health evaluation II; SOFA, sequential organ failure assessment; CRRT, Continuous Renal Replacement Therapy; ECMO, Extracorporeal Membrane Oxygenation. * on ICU admission day.

**Table 2:** Area under the receiver operating characteristics curve for 28-day mortality in the study participants.

Variable	AUC	SE	*p*-Value	95% CI	Cutoff	Sensitivity	Specificity	AUC Difference	*p*-Value (DeLong Test)
Granzyme B	0.794	0.060	<0.001	0.676–0.913	41.75	0.840	0.750	Reference	
Lactate	0.804	0.070	<0.001	0.668–0.941	6.7	0.591	1.000	−0.010	0.778
SOFA	0.764	0.069	<0.001	0.629–0.898	13.5	0.640	0.889	0.030	0.956
APACHE II	0.709	0.076	0.006	0.560–0.857	31.5	0.640	0.781	0.085	0.372
IL-6	0.604	0.079	0.910	0.449–0.758	1048.1	0.560	0.719	0.190	0.009
Lactate + Granzyme B	0.838	0.061	<0.001	0.718–0.958	43.7	0.864	0.783	−0.044	0.252

SOFA, sequential organ failure assessment; APACHE II, acute physiology and chronic health evaluation II; AUC, area under the curve; SE, standard error; CI, confidence interval; IL-6, interleukin-6.

**Table 3:** Cox proportional hazards regression analysis for 28-day mortality.

	Univariable			Multivariable		
Variables	HR	95% CI	*p*-Value	HR	95% CI	*p*-Value
Age	1	0.97–1.03	0.979	0.98	0.96–1.01	0.275
Male (vs. Female)	1.13	0.50–2.55	0.776			
Charlson comorbidity index	1.26	1.08–1.47	0.003	1.18	1.02–1.36	0.031
Malignancy	2.6	1.18–5.73	0.018			
APACHE II score	1.07	1.02–1.12	0.003			
Total SOFA score	1.21	1.09–1.34	<0.001	1.08	0.96–1.22	0.209
C-reactive protein (mg/L)	1	0.99–1.00	0.055			
Lactate, venous (mmol/L)	1.21	1.12–1.31	<0.001			
Granzyme B ≥ 41.75 pg/mL	8.16	2.77–24.02	<0.001	4.47	1.29–15.44	0.018

HR, hazard ratio; CI, confidence interval; APACHE II, acute physiology and chronic health evaluation score II; SOFA, sequential organ failure assessment.

## Data Availability

The raw data supporting the conclusions of this article will be made available by the authors on request.
